# Pasireotide LAR maintains inhibition of GH and IGF-1 in patients with acromegaly for up to 25 months: results from the blinded extension phase of a randomized, double-blind, multicenter, Phase III study

**DOI:** 10.1007/s11102-014-0585-6

**Published:** 2014-08-08

**Authors:** Michael Sheppard, Marcello D. Bronstein, Pamela Freda, Omar Serri, Laura De Marinis, Luciana Naves, Liudmila Rozhinskaya, Karina Hermosillo Reséndiz, Matthieu Ruffin, YinMiao Chen, Annamaria Colao

**Affiliations:** 1 Centre for Endocrinology, Diabetes and Metabolism, University of Birmingham, Edgbaston, Birmingham, B15 2TT UK; 2 Neuroendocrine Unit, Division of Endocrinology and Metabolism, University of São Paulo Medical School, São Paulo, Brazil; 3 Department of Medicine, Columbia University College of Physicians and Surgeons, New York, NY USA; 4 Service of Endocrinology, Centre Hospitalier de l’Université de Montréal, Notre-Dame Hospital, University of Montreal, Montreal, Canada; 5 Section of Endocrinology, Department of Internal Medicine, Università Cattolica del Sacro Cuore, Rome, Italy; 6 Unit of Endocrinology, Department of Internal Medicine, University of Brasilia, Brasilia, Brazil; 7 Endocrinology Research Center, Moscow, Russia; 8 Clinical Development, Novartis Pharmaceuticals Corporation, Florham Park, NJ USA; 9 Clinical Development, Oncology Business Unit, Novartis Pharma AG, Basel, Switzerland; 10 Novartis Pharmaceuticals Corporation, East Hanover, NJ USA; 11 Dipartimento di Medicina Clinica e Chirurgia, Università Federico II di Napoli, Naples, Italy

**Keywords:** Pasireotide, Octreotide, Acromegaly, Extension

## Abstract

**Purpose:**

A large, randomized, double-blind, Phase III core study demonstrated that pasireotide LAR was significantly superior to octreotide LAR at providing GH <2.5 μg/L and normalized IGF-1 after 12 months’ treatment in patients with acromegaly. We report the efficacy and safety of pasireotide LAR and octreotide LAR after up to 26 months’ treatment.

**Methods:**

Patients with GH <2.5 μg/L and IGF-1 ≤1× ULN at month 12, or patients considered to be experiencing clinical benefit, were eligible to continue receiving their randomized therapy in the extension. Efficacy and safety in the pasireotide LAR and octreotide LAR groups were evaluated for up to 26 months.

**Results:**

Overall, 120 patients who completed the core study continued receiving pasireotide LAR (n = 74) or octreotide LAR (n = 46) in the extension. At month 25, biochemical control (GH <2.5 μg/L and normal IGF-1) was achieved by 48.6 % (36/74) and 45.7 % (21/46) of patients in the pasireotide LAR and octreotide LAR arms [60.8 % (45/74) and 52.2 % (24/46) when including patients with IGF-1 < LLN], respectively. In total, 74.7 % of pasireotide LAR and 71.6 % of octreotide LAR patients had tumor volume decrease ≥20 % from baseline to month 26. Most AEs were mild or moderate. Hyperglycemia-related AEs were seen in 62.9 and 25.0 % of pasireotide LAR and octreotide LAR patients, respectively. No new safety signals were observed in the extension compared with the core study.

**Conclusions:**

GH and IGF-1 suppression is maintained for up to 25 months during pasireotide LAR treatment. The safety profile of pasireotide LAR is typical of a somatostatin analogue, except for the frequency and degree of hyperglycemia.

**Electronic supplementary material:**

The online version of this article (doi:10.1007/s11102-014-0585-6) contains supplementary material, which is available to authorized users.

## Introduction

Acromegaly is characterized by chronic hypersecretion of growth hormone (GH), which primarily originates from a GH-secreting pituitary adenoma and induces the synthesis of insulin-like growth factor 1 (IGF-1). Elevated GH and IGF-1 levels cause metabolic dysfunction and somatic growth, resulting in significant morbidity and mortality for patients with acromegaly. As decreasing GH to <2.5 μg/L *and* IGF-1 to normal levels significantly reduces mortality [1–4], the main treatment goal for acromegaly is to control GH and IGF-1 levels. Additional goals are to reduce and/or stabilize tumor size, preserve pituitary function and prevent recurrence [5].

Long-acting somatostatin analogues are the cornerstone of medical therapy for acromegaly and are indicated in patients with failed surgery or as first-line treatment when surgery is contraindicated or declined [6, 7]. Various studies have demonstrated that somatostatin analogues can be effective as both first- and second-line therapy over long-term treatment [8–13]. In selected patients with active acromegaly, long-term somatostatin analogue therapy has been demonstrated to effectively control GH and IGF-1 levels, induce tumor volume reduction and improve hypertension and cardiac performance [9, 10, 12]. However, many patients with acromegaly do not achieve biochemical control with the currently available somatostatin analogues [14].

Pasireotide has a broader somatostatin receptor (sst) binding profile than the currently available somatostatin analogues. Pasireotide has 30-, 5- and 39-fold higher binding affinity for sst_1_, sst_3_ and sst_5_, respectively, than octreotide, and a slightly lower affinity for sst_2_ [15]. As demonstrated previously, pasireotide has the potential to be an effective medical therapy for patients with acromegaly [16, 17]. In a large, randomized, 12-month, Phase III core study in medically naïve patients with acromegaly, pasireotide long-acting release (LAR) was significantly superior (*P* = 0.007) to octreotide LAR at providing GH <2.5 μg/L and normal IGF-1 [16]. Patients who completed this core study and had GH <2.5 μg/L and IGF-1 ≤1× the upper limit of normal (ULN) at month 12, or who were considered by the investigator to be receiving clinical benefit (irrespective of biochemical control), were able to continue on their randomized therapy in a planned extension study. Here we report the efficacy and safety of pasireotide LAR and octreotide LAR after up to 26 months’ treatment in these patients.

## Methods

### Study design and patient population

This was a double-blind extension to a multicenter, 12-month, Phase III core study enrolling medically naïve patients with active acromegaly who either had prior pituitary surgery or were de novo with a visible pituitary adenoma on magnetic resonance imaging (MRI) [16]. In the 12-month core study, patients were randomized to pasireotide LAR 40 mg every 28 days (n = 176) or octreotide LAR 20 mg every 28 days (n = 182) [16]. A dose increase to pasireotide LAR 60 mg or octreotide LAR 30 mg was permitted, but not mandatory, at month 3 or 7 based on biochemical response (mean GH ≥2.5 µg/L and/or IGF-1 >1× ULN). Dose decreases (pasireotide LAR: from 60 to 40 mg or from 40 to 20 mg; octreotide LAR: from 30 to 20 mg or from 20 to 10 mg) were permitted for tolerability, as was an increase to the original dose upon resolution. A detailed description of the methods and dosing employed in the core study has been published [16].

**Fig. 1:**
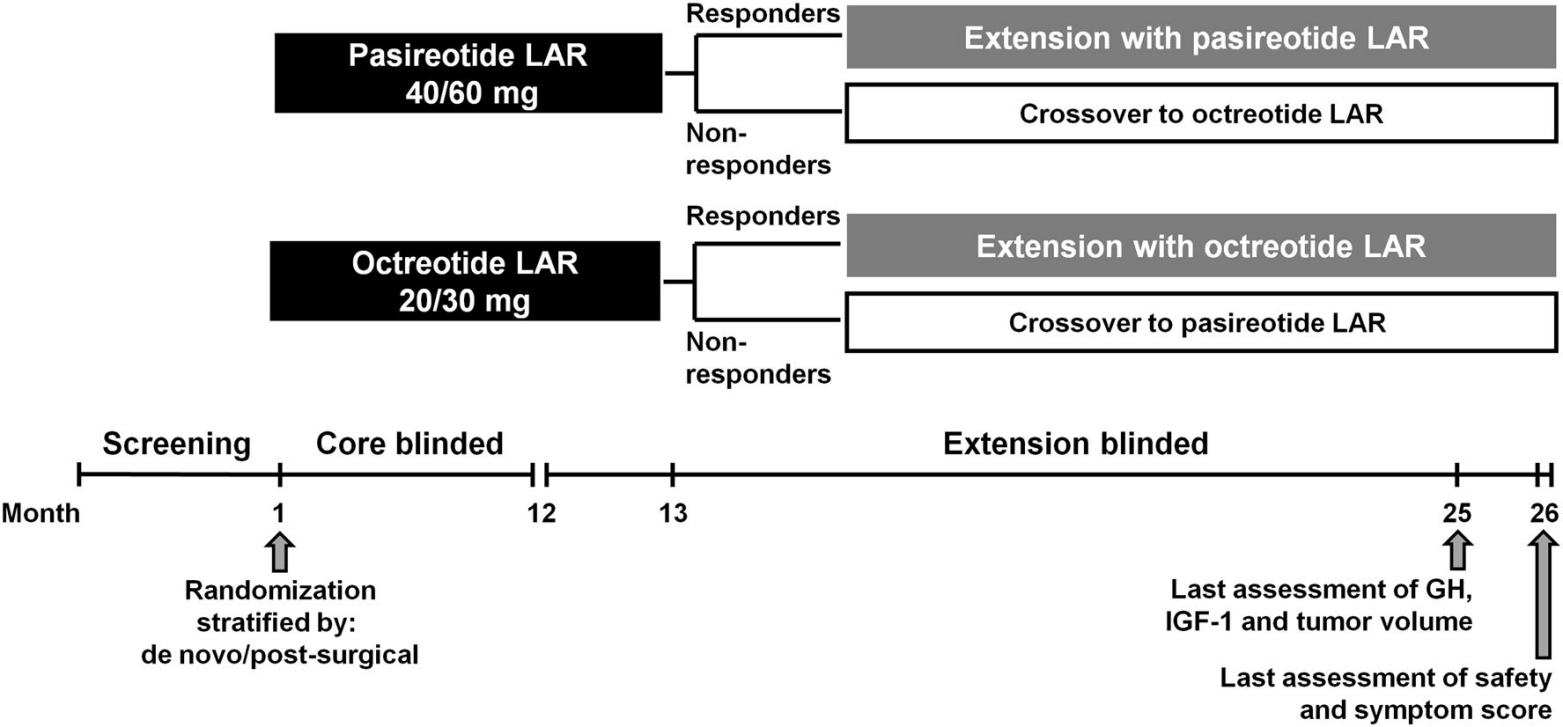
Study design

In the extension phase, patients with GH <2.5 μg/L and IGF-1 ≤1× ULN at month 12, or those patients considered by the investigator to be experiencing clinical benefit from the study drug, were eligible to continue receiving their randomized therapy (Fig. 1). Patients with GH ≥2.5 μg/L and/or IGF-1 >1× ULN could switch treatment to either pasireotide LAR 40 mg every 28 days or octreotide LAR 20 mg every 28 days at month 13; results from these patients are not reported here and will be described in a separate manuscript. Patients continuing on their randomized therapy received the same pasireotide LAR or octreotide LAR dose in the extension phase that they were receiving at the end of the core study. A dose increase was permitted at any time during the extension when GH levels were ≥2.5 µg/L and/or IGF-1 >1× ULN; maximum permitted doses of pasireotide LAR and octreotide LAR were 60 and 30 mg every 28 days, respectively. Dose decreases as with the core study were permitted at any time in the event of tolerability issues; the dose could be increased to the original dose upon resolution.

Thirty-four patients entered the extension phase before a protocol amendment was implemented; these patients received unblinded pasireotide LAR treatment during the extension. Of these patients, 15 received pasireotide LAR and 19 received octreotide LAR during the core study. Prior to the protocol amendment, patients randomized to octreotide LAR who achieved GH <2.5 μg/L and IGF-1 ≤1× ULN at month 12 could not continue receiving octreotide LAR during the extension phase; these patients were considered to have completed the study and were discontinued. Additionally, patients randomized to pasireotide LAR did not have the option to switch to octreotide LAR at month 13 if GH was ≥2.5 μg/L and/or IGF-1 >1× ULN. Following the protocol amendment, patients in both treatment arms who had GH <2.5 μg/L and IGF-1 ≤1× ULN or were considered by the investigator to be achieving drug-related clinical benefit at month 12 could continue on their randomized therapy, and patients in both treatment arms could switch therapies at month 13. Patients who entered the extension phase after the protocol amendment was implemented received treatment in a double-blind manner up to month 26. Nineteen patients in the pasireotide LAR arm and 13 patients in the octreotide LAR arm completed the core study prior to the protocol amendment but did not enter the extension phase. Based on manual review of the response status for these patients at month 12, it is estimated that four patients in the octreotide LAR arm would have potentially been eligible to continue receiving octreotide LAR during the extension phase had they reached the end of the core study after the protocol amendment was implemented; 14 patients in the pasireotide LAR arm would have potentially been eligible to cross over to octreotide LAR treatment during the extension phase.

The study was conducted in accordance with the Declaration of Helsinki, and an independent ethics committee or institutional review board for each study site approved the study protocol. All patients provided written informed consent to participate in the trial. The ClinicalTrials.gov identifier is NCT00600886.

### Endpoints and assessments

The key efficacy endpoint assessed was the proportion of patients who achieved biochemical control, defined as GH <2.5 μg/L (mean GH concentration of a five-point profile within a 2-hour time period) *and* normal IGF-1 (according to age and sex), at the end of the blinded extension phase. Additional efficacy endpoints were the proportion of patients achieving GH <2.5 μg/L *and* IGF-1 ≤1× ULN (i.e. including patients who had IGF-1 levels below the lower limit of normal [LLN]), the proportion achieving GH <2.5 µg/L, the proportion achieving normal IGF-1, and overall changes in GH and IGF-1 levels. All GH and IGF-1 samples were measured using validated chemiluminescent immunometric assays [Immulite^®^ 2000/1000; Diagnostic Products Corp (Siemens), Los Angeles, CA; GH International Reference Preparation (IRP) WHO NIBSC 2nd IS 98/574; IGF-1 IRP WHO NIBSC 1st IRR 87/518]. The lower limit of detection for GH was 0.1 μg/L, with intra- and inter-assay coefficients of variation ≤6.6 %. For IGF-1, the lower limit of detection was 20 μg/L, with intra- and inter-assay coefficients of variation ≤6.7 %. IGF-1 values were compared with age- and sex-standardized normal values. All samples except for those from China were analyzed using the Immulite^®^ 2000 assay by Quest Diagnostics Nichols Institute Laboratory, San Juan Capistrano, California, USA between March 2008 and March 2010, then by Quest Diagnostics Clinical Trials Laboratory, Valencia, California, USA from March 2010 onwards. Samples from China were analyzed using the Immulite^®^ 1000 assay by Kingmed Diagnostics, Guangzhou, China. Subsequent to receiving a notification from Siemens regarding the Immulite^®^ IGF-1 assay, Quest Diagnostics reviewed all quality control data collected from the three laboratories and verified that all IGF-1 data are valid.

Tumor volume was evaluated every 6 months by gadolinium-enhanced pituitary MRI performed by a central reader blinded to treatment. A pituitary tumor volume change of ≥20 % from screening was considered significant. Tumor volume was calculated by hand-drawing around the tumor circumference in coronal cross-sections, multiplying the area by slice thickness, and summing the resulting volumes across all slices containing tumor. As the last assessment of GH, IGF-1 and tumor volume prior to the end of the planned blinded extension phase was performed at month 25, these data are reported up to month 25. Changes in five signs and symptoms of acromegaly (headache, fatigue, perspiration, paresthesia and osteoarthralgia) were evaluated monthly and scored from 0 (no symptom) to 4 (very severe); data are presented up to month 26. See the Supplementary Appendix for further details.

Safety was assessed based on the monitoring and recording of all adverse events (AEs), evaluated according to the National Cancer Institute Common Terminology Criteria for AEs version 3.0 [18], as well as regular monitoring of hematology, blood chemistry and urinalysis parameters, performance of physical examinations and electrocardiograms, and body weight measurements. Blood samples for laboratory tests, including blood glucose measurements, were drawn at each visit under fasted conditions before the morning dose of study drug. Safety data are presented for all patients in the core study (i.e. up to month 12) and all patients who continued on their randomized therapy in the extension phase (i.e. months 12–26). Extension-phase data from patients who switched treatments at the end of the core study are not included and will be published separately.

### Statistical analyses

This analysis focuses on data from patients who continued on their randomized therapy in the extension phase. If <3 GH samples were taken for the assessment of mean GH (based on a five-point 2-hour profile), the value was considered to be missing at the corresponding visit. If GH and IGF-1 assessments were taken >35 days after the pasireotide LAR or octreotide LAR injection, these values were also considered to be missing at the corresponding visit. The calculation of the proportion of patients who achieved GH <2.5 μg/L *and* normal IGF-1 during the extension phase was performed without the imputation of missing values. Patients who discontinued treatment were considered to be non-responders at later time points. If one of the two biochemical values (mean GH or IGF-1) was missing, and if the available value did not meet the response criteria for the given parameter, the patient was considered to be a non-responder at that visit; if the available value met the response criteria set for the given biochemical parameter, the patient’s response status was considered missing. Descriptive statistics for quantitative variables (i.e. GH, IGF-1, tumor volume, signs and symptoms scores) were based on available measurements. No formal statistical analyses were planned to compare the pasireotide LAR and octreotide LAR treatment arms during the extension phase. In addition, the extension phase was not powered to determine a difference in efficacy or safety outcomes between the two treatment arms. For the purposes of this analysis, changes in patient diabetic status were calculated according to current American Diabetes Association (ADA) criteria for normal glucose tolerance [glycated hemoglobin (HbA_1c_) <5.7 %], pre-diabetes (HbA_1c_ ≥5.7 to <6.5 %) and diabetes (HbA_1c_ ≥6.5 %).

## Results

### Patient population

**Fig. 2:**
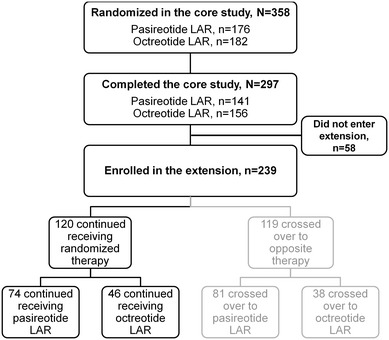
Flowchart showing the number of patients who were randomized, completed the 12-month core study and entered the extension phase

In total, 141/176 (80.1 %) and 156/182 (85.7 %) patients in the pasireotide LAR and octreotide LAR arms, respectively, completed the 12-month core study. Of those patients who completed the core study, 74 pasireotide LAR patients and 46 octreotide LAR patients continued on their randomized therapy in the extension phase (Table 1; Fig. 2). The mean duration of exposure to study treatment was 527 days (1.4 years) in the pasireotide LAR arm and 415 days (1.1 years) in the octreotide LAR arm.

**Table 1:** Patient demographics and characteristics at core baseline for patients who continued on their randomized therapy in the extension phase

Demographic variable	Pasireotide LAR (n = 74)	Octreotide LAR (n = 46)
Age, years	*n* = *74*	*n* = *46*
Median (range)	46.5 (22 − 71)	45.0 (24 − 70)
Sex, n (%)		
Female	38 (51.4)	26 (56.5)
Male	36 (48.6)	20 (43.5)
Race, n (%)		
Caucasian	41 (55.4)	28 (60.9)
Black	0	1 (2.2)
Asian	21 (28.4)	9 (19.6)
Native American	3 (4.1)	0
Other	9 (12.2)	8 (17.4)
Previous surgery, n (%)	37 (50.0)	20 (43.5)
GH level, μg/L	*n* = *70*	*n* = *45*
Mean (SD)	14.4 (21.4)	11.3 (12.7)
Median (range)	6.9 (0.8–114.6)	6.5 (1.4–64.1)
IGF-1 level, × ULN	*n* = *74*	*n* = *46*
Mean (SD)	2.6 (1.2)	2.5 (1.1)
Median (range)	2.4 (0.9–5.9)	2.4 (0.9–6.5)
Tumor volume, mm^3^	*n* = *71*	*n* = *43*
Mean (SD)	1,981 (4,515)	2,429 (4,936)
Median (range)	646 (0–35,095)	772 (53–25,473)

### Effect of treatment on GH and IGF-1 levels

Of the patients who continued receiving their randomized treatment in the extension phase, 53/74 (71.6 %) in the pasireotide LAR arm and 26/46 (56.5 %) in the octreotide LAR arm had GH <2.5 μg/L *and* IGF-1 ≤1× ULN at entry into the extension phase. The remainder were judged by the investigator to have been receiving clinical benefit from treatment. Of these patients, seven in the pasireotide LAR arm and 11 in the octreotide LAR arm were partial responders (defined as GH <5 µg/L *and* IGF-1 ≤1.3× ULN, but not biochemical control), while 13 and eight patients, respectively, were non-responders (defined as neither a responder nor a partial responder), at entry into the extension phase. This information was missing from one patient in each group.

**Fig. 3:**
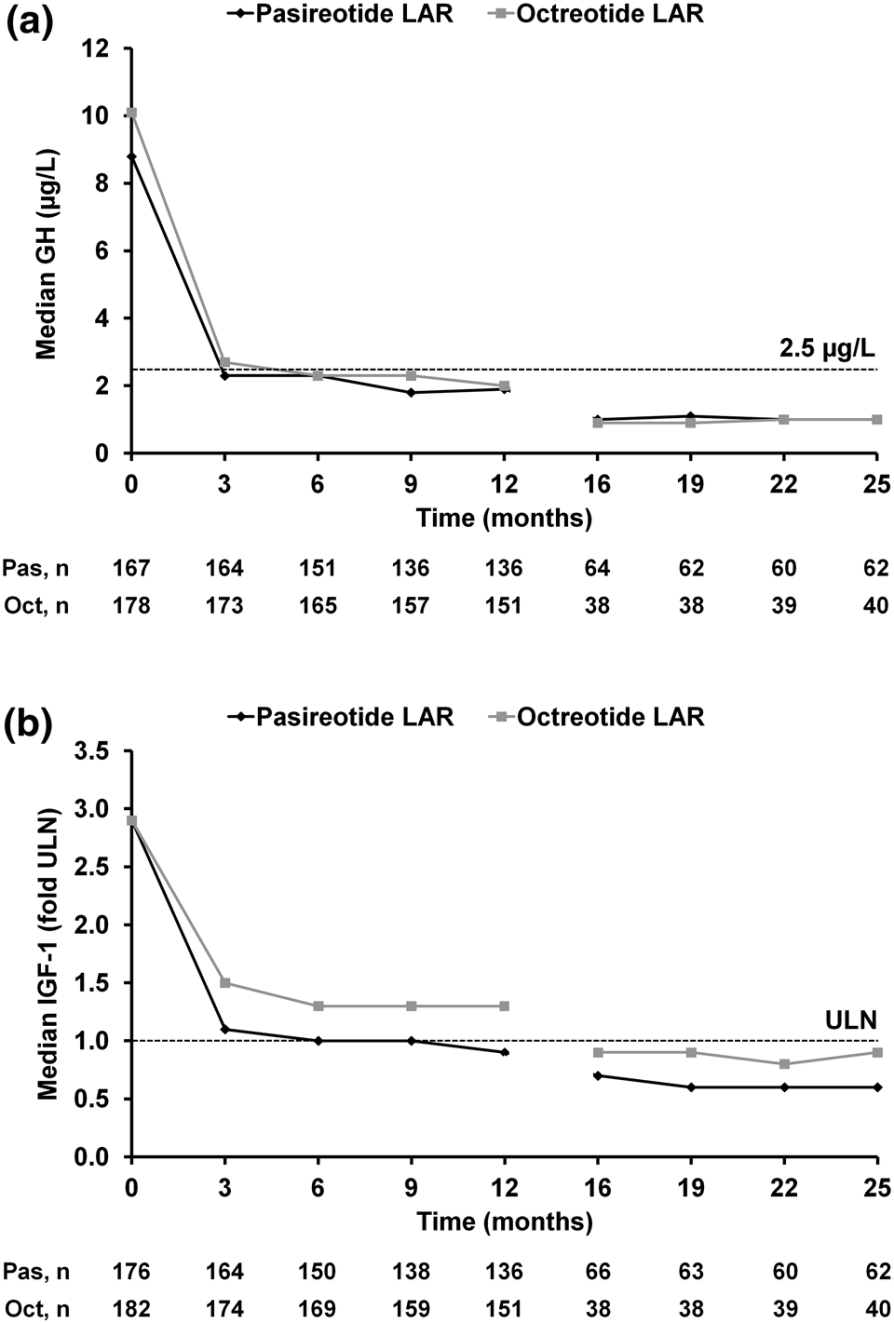
Median **a** GH and **b** IGF-1 levels during treatment. The total numbers of patients with evaluable measurements for GH and IGF-1 are shown beneath each graph. *Oct* octreotide LAR; *Pas* pasireotide LAR

At month 25, 36/74 (48.6 %) pasireotide LAR and 21/46 (45.7 %) octreotide LAR patients had biochemical control (GH <2.5 μg/L *and* normal IGF-1), while 45/74 (60.8 %) and 24/46 (52.2 %) had mean GH <2.5 μg/L and IGF-1 ≤1× ULN (Table 2). The proportion of patients with a reduction in mean GH to <2.5 μg/L at the end of the extension phase was 70.3 % (n = 52/74) for the pasireotide LAR and 80.4 % (n = 37/46) for the octreotide LAR arms (Table 2). Overall, 51.4 % (38/74) and 47.8 % (22/46) of patients in the pasireotide LAR and octreotide LAR arms, respectively, had normal IGF-1 at the end of the extension (Table 2). Median GH levels were <2.5 μg/L by month 3 in the pasireotide LAR arm and by month 6 in the octreotide LAR arm and remained below this threshold up to month 25. Median IGF-1 levels were within the normal range by month 6 in the pasireotide LAR arm and month 16 in the octreotide LAR arm and remained so at month 25 (Fig. 3).

**Table 2:** Biochemical response rates

	Pasireotide LAR (n = 74)		Octreotide LAR (n = 46)	
	n/N (%)	95 % CI	n/N (%)	95 % CI
GH <2.5 μg/L *and* IGF-1 normalization				
Month 12	46/74 (62.2)	50.1, 73.2	24/46 (52.2)	36.9, 67.1
Month 19	34/74 (45.9)	34.3, 57.9	21/46 (45.7)	30.9, 61.0
Month 25	36/74 (48.6)	36.9, 60.6	21/46 (45.7)	30.9, 61.0
GH <2.5 μg/L *and* IGF-1 ≤1× ULN				
Month 12	53/74 (71.6)	59.9, 81.5	26/46 (56.5)	41.1, 71.1
Month 19	44/74 (59.5)	47.4, 70.7	23/46 (50.0)	34.9, 65.1
Month 25	45/74 (60.8)	48.8, 72.0	24/46 (52.2)	36.9, 67.1
GH <2.5 μg/L				
Month 12	58/74 (78.4)	67.3, 87.1	37/46 (80.4)	66.1, 90.6
Month 19	54/74 (73.0)	61.4, 82.6	33/46 (71.7)	56.5, 84.0
Month 25	52/74 (70.3)	58.5, 80.3	37/46 (80.4)	66.1, 90.6
IGF-1 normalization				
Month 12	55/74 (74.3)	62.8, 83.8	26/46 (56.5)	41.1, 71.1
Month 19	37/74 (50.0)	38.1, 61.9	24/46 (52.2)	36.9, 67.1
Month 25	38/74 (51.4)	39.4, 63.1	22/46 (47.8)	32.9, 63.1

Patients who discontinued treatment during the extension phase were considered to be non-responders at subsequent time points

### Tumor response

The mean decrease from core baseline to the end of the blinded extension phase was 600 ± 735 mm^3^ (51.8 % decrease) and 1,120 ± 2,541 mm^3^ (55.0 % decrease) in the pasireotide LAR and octreotide LAR arms, respectively. The proportion of patients who achieved a significant reduction in tumor volume of ≥20 % at the end of the blinded extension phase was similar in both treatment groups (74.7 % for pasireotide LAR and 71.6 % for octreotide LAR); the median time to significant tumor volume reduction was 25.0 weeks for pasireotide LAR and 24.3 weeks for octreotide LAR.

### Effect on signs and symptoms of acromegaly

The five assessed symptoms of acromegaly improved from core baseline in both treatment groups. By month 26, decreases in mean symptom scores from core baseline were seen in the pasireotide LAR and octreotide LAR arms for headache (0.0 ± 0.9 and 0.2 ± 0.9), fatigue (0.4 ± 1.4 and 0.4 ± 1.4), perspiration (0.0 ± 0.7 and 1.0 ± 1.2), osteoarthralgia (0.3 ± 1.2 and 0.2 ± 1.3), and paresthesia (0.2 ± 0.6 and 0.3 ± 0.8).

### Safety and tolerability during core and extension

Overall, 23/74 (31.1 %) and 10/46 (21.7 %) patients in the pasireotide LAR and octreotide LAR arms who continued receiving their randomized treatment in the extension phase discontinued treatment between months 12 and 26. Of these, 11/23 (47.8 %) patients in the pasireotide LAR arm and 3/10 (30.0 %) patients in the octreotide LAR arm had GH <2.5 μg/L and IGF-1 ≤1× ULN at last assessment. The most common reason for discontinuation during the blinded extension phase was consent withdrawal (12.2 and 4.3 % in the pasireotide LAR and octreotide LAR arms, respectively); review of these cases revealed that the majority of patients who withdrew consent did so because they elected to undergo pituitary surgery. During the blinded extension phase, two (2.7 %) patients in the pasireotide LAR arm and one (2.2 %) patient in the octreotide LAR arm discontinued because of AEs; one of these patients, who was receiving pasireotide LAR, discontinued because of a serious AE. Two deaths occurred during the extension phase (major depression leading to suicide in a pasireotide LAR patient, sepsis in an octreotide LAR patient). Neither of the deaths were considered by the investigator to be related to study drug.

Most patients experienced at least one AE with a suspected relationship to study drug during the 26-month study period (86.5 and 77.2 % in the pasireotide LAR and octreotide LAR arms, respectively); most were mild to moderate in nature. The most common AEs were diarrhea and cholelithiasis (Table 3). Overall, 112 patients (62.9 %) in the pasireotide LAR arm experienced a hyperglycemia-related AE (all terms relating to elevations in blood glucose, e.g. hyperglycemia, diabetes mellitus, etc.), compared with 45 patients (25.0 %) in the octreotide LAR arm. In total, 32 (18.0 %) and 27 (15.0 %) patients experienced at least one serious AE in the pasireotide LAR and octreotide LAR arms, respectively; 11 (6.2 %) and 11 (6.1 %) patients experienced at least one serious drug-related AE.

**Table 3:** Most common AEs (>10 % in either treatment arm) reported during the 26-month study period, regardless of relationship to study drug

	Pasireotide LAR (N = 178^a^)		Octreotide LAR (N = 180^a^)	
	All grades	Grade 3/4	All grades	Grade 3/4
	n (%)	n (%)	n (%)	n (%)
Diarrhea	71 (39.9)	1 (0.6)	81 (45.0)	5 (2.8)
Hyperglycemia	55 (30.9)	6 (3.4)	18 (10.0)	1 (0.6)
Cholelithiasis	54 (30.3)	2 (1.1)	71 (39.4)	3 (1.7)
Headache	40 (22.5)	2 (1.1)	49 (27.2)	5 (2.8)
Diabetes mellitus	38 (21.3)	9 (5.1)	8 (4.4)	0
Alopecia	34 (19.1)	0	36 (20.0)	0
Abdominal pain	33 (18.5)	1 (0.6)	44 (24.4)	0
Nasopharyngitis	31 (17.4)	0	29 (16.1)	0
Nausea	27 (15.2)	1 (0.6)	41 (22.8)	0
Increased blood creatine phosphokinase	25 (14.0)	6 (3.4)	24 (13.3)	4 (2.2)
Arthralgia	21 (11.8)	1 (0.6)	25 (13.9)	1 (0.6)
Back pain	21 (11.8)	0	22 (12.2)	2 (1.1)
Abdominal distension	21 (11.8)	1 (0.6)	22 (12.2)	1 (0.6)
Dizziness	21 (11.8)	0	20 (11.1)	0
Fatigue	19 (10.7)	1 (0.6)	21 (11.7)	0
Hypertension	18 (10.1)	2 (1.1)	16 (8.9)	4 (2.2)
Constipation	10 (5.6)	0	19 (10.6)	0

AEs are presented in overall descending order for the pasireotide LAR arm, starting with the most frequent

^a^Two patients randomized to the octreotide LAR treatment arm received pasireotide LAR in error. These two patients are included in the pasireotide LAR treatment arm for the purposes of the safety analysis

**Fig. 4:**
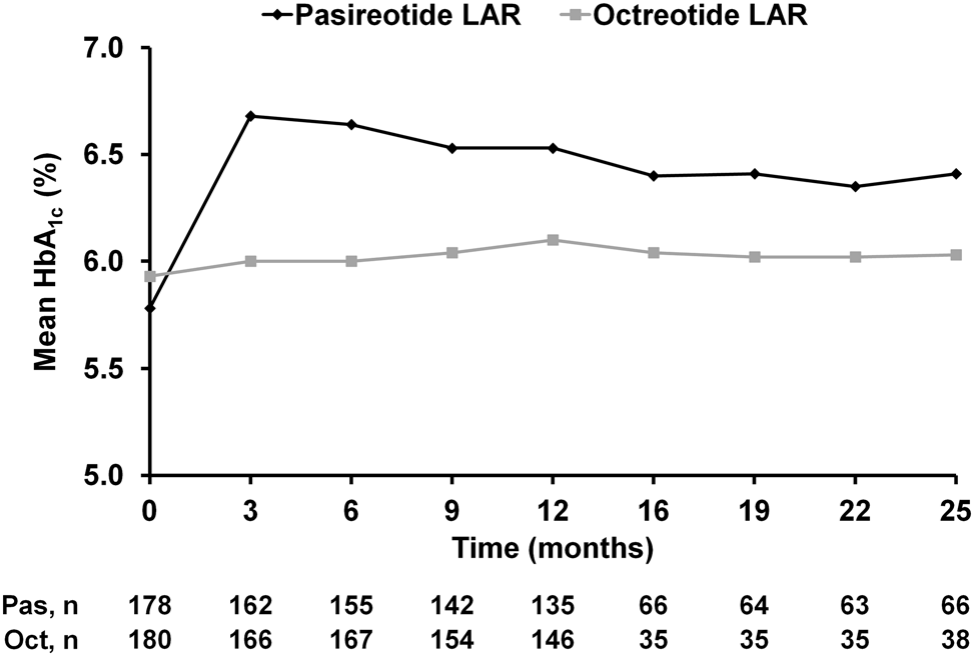
Mean HbA_1c_ over time from core baseline up to month 25

Mean glucose and HbA_1c_ increased in the first 3 months after initiation of pasireotide therapy and remained stable to 26 months (Fig. 4). For octreotide, a smaller and more gradual increase in mean glucose and HbA_1c_ was observed, which peaked at months 9–12 and remained relatively stable up to month 26. Of the 119 patients in the pasireotide LAR arm with normal fasting glucose (<100 mg/dL) at core baseline, 83 (69.7 %) experienced worsening of fasting glucose levels at last available assessment during the core or extension; 23/51 (45.1 %) patients who had core baseline levels of 100 to <126 mg/dL had levels ≥126 mg/dL at the last available assessment (Table 4). In the octreotide LAR arm, 43/109 (39.4 %) patients with normal baseline fasting glucose levels and 7/54 (13.0 %) patients with baseline levels of 100 to <126 mg/dL had worse levels at the last available assessment.

**Table 4:**
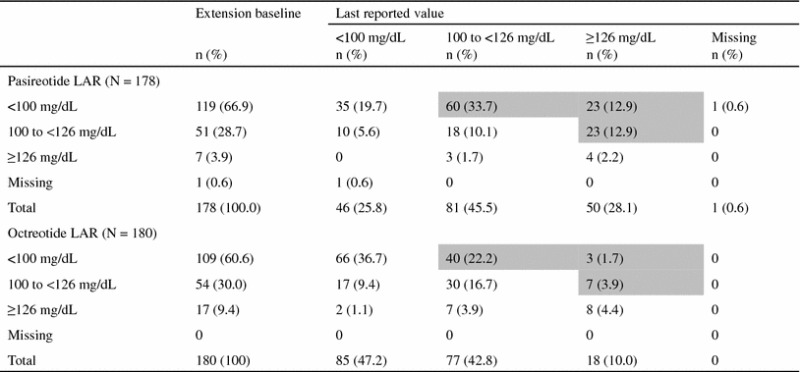
Overall shift in glucose from core baseline to last available value up to month 26

Shaded boxes represent the patients with a shift in glucose level (mg/dL) that indicates a worse diabetic status at the last reported value compared with core baseline

At baseline, 41.6 % (74/178) of patients in the pasireotide LAR arm had normal HbA_1c_ levels; 52.7 % (39/74) and 23.0 % (17/74) of these patients had last available HbA_1c_ levels in the pre-diabetic and diabetic ranges, respectively (Table 5). Overall, 58.4 % (104/178) of patients in the pasireotide LAR arm had a last available HbA_1c_ level indicating a worse diabetic status than at core baseline. Sixty-six patients in the octreotide LAR arm had normal HbA_1c_ levels at baseline. Of these patients, 32 (48.5 %) and two (3.0 %) had last available HbA_1c_ levels in the pre-diabetic and diabetic ranges, respectively. Overall, 28.3 % (51/180) of patients in the octreotide LAR arm had a last available HbA_1c_ level indicating a worse diabetic status than at core baseline.

**Table 5:**
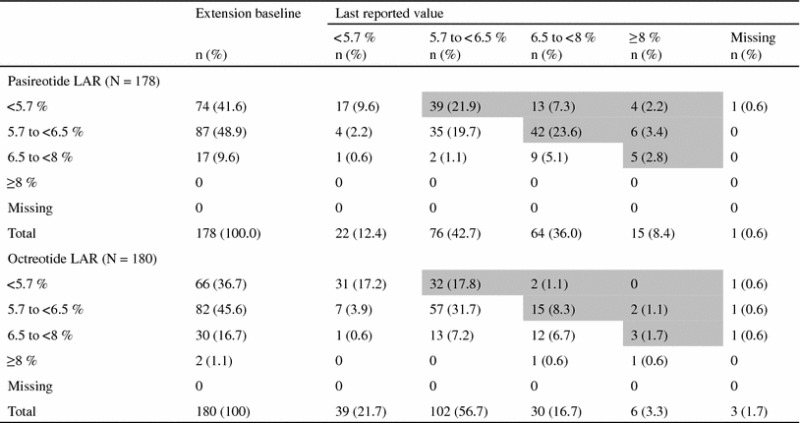
Overall shift in HbA_1c_ from core baseline to last available value up to month 26

Shaded boxes represent the patients with a shift in HbA_1c_ level (%) that indicates a worse diabetic status at the last reported value compared with core baseline

In total, 85 (47.8 %) pasireotide LAR patients and 48 (26.7 %) octreotide LAR patients received concomitant anti-diabetic medication during the 26-month study period; metformin was most commonly used. Of the patients who were not receiving anti-diabetic medication at core baseline, at least one anti-diabetic medication was initiated in 40.4 % (63/156) of patients in the pasireotide LAR arm and 5.7 % (8/140) of patients in the octreotide LAR arm.

## Discussion

This report from the extension phase of a large Phase III study is the first long-term analysis of pasireotide LAR in medically naïve patients (42 % post-surgical and 58 % de novo at core baseline) with acromegaly. It demonstrates that GH and IGF-1 suppression is maintained in patients who continue receiving pasireotide LAR and octreotide LAR for up to 25 months and that long-term treatment is generally well tolerated. Importantly, median GH levels were <2.5 μg/L and IGF-1 levels were maintained below the ULN for up to 25 months in both treatment arms. In addition to effects on GH and IGF-1, both pasireotide LAR and octreotide LAR provided overall clinical benefit by effectively improving the signs and symptoms of acromegaly and reducing tumor volume. These results are in line with those of previous long-term studies of somatostatin analogues in acromegaly [8–13]; however, few have evaluated a combined clinical endpoint of both GH <2.5 μg/L *and* normalization of IGF-1.

Patients were eligible to enter the extension study if they had achieved biochemical control in the 12-month core study, or if the investigator judged that they were receiving clinical benefit from treatment (irrespective of biochemical control). Fewer patients in the octreotide LAR arm were eligible to continue on their randomized therapy in the extension phase compared with the pasireotide LAR arm (25.3 versus 42.0 % of patients, respectively). This is in line with the core study data showing that pasireotide LAR was significantly superior to octreotide LAR at providing GH <2.5 μg/L *and* normal IGF-1 [16], even after accounting for the fact that four patients could not continue receiving octreotide LAR in the extension phase as they entered the study before the protocol amendment was implemented. As the extension phase was not designed or powered to compare pasireotide LAR with octreotide LAR, no conclusions can be drawn regarding the relative efficacy of these somatostatin analogues over the 25-month treatment period. In addition, because all patients entering the extension were benefiting from their randomized treatment, no difference between the treatment arms would be expected.

At the beginning of the extension phase, 71.6 % of patients in the pasireotide LAR arm and 56.5 % in the octreotide LAR arm had GH <2.5 μg/L and IGF-1 ≤1× ULN. After 25 months of treatment, the proportions were 60.8 and 52.2 %, respectively. The most common reason for discontinuation in both treatment arms during the blinded extension phase was consent withdrawal; the majority of these patients elected to undergo pituitary surgery.

During the blinded extension phase, the safety profile of pasireotide LAR was similar to that of octreotide LAR, except for the frequency and degree of hyperglycemia. The safety profiles of pasireotide LAR and octreotide LAR during the blinded extension phase were similar to those seen in the 12-month core study. However, although HbA_1c_ and glucose levels initially increased in the first 3 months of pasireotide treatment, they then remained stable to 26 months. The administration of anti-diabetic medication, most commonly metformin, may have contributed to the lack of further deterioration in glucose homeostasis with pasireotide LAR; almost half of patients receiving pasireotide LAR received an anti-diabetic agent during the 26-month study, while ≥1 agent was initiated in 40.4 % of patients who were not receiving medication at baseline. As changes in the dose of concomitant medications were not recorded during the 26 months of treatment, further studies are necessary to determine the effects of anti-diabetic therapies on the stabilization of HbA_1c_ and glucose levels during pasireotide treatment. As sst_5_ receptors are expressed on tissues such as pancreatic islet cells [19], the higher levels of hyperglycemia observed with pasireotide may be a result of its higher binding affinity for sst_5_ compared with other somatostatin analogues. A recent study in healthy volunteers has shown that although pasireotide inhibits insulin secretion and incretin response, with modest suppression of glucagon, it does not affect insulin sensitivity [20]. After initiation of pasireotide, all patients should be closely monitored for changes in glucose homeostasis; in the event of hyperglycemia, prompt intervention is warranted. Importantly, no new safety signals were identified in the extension phase that would caution against long-term use of pasireotide LAR.

In conclusion, pasireotide LAR and octreotide LAR provide long-term inhibition of GH and IGF-1 in patients with acromegaly. Therefore, pasireotide LAR is a viable new treatment option for patients with acromegaly.

## Electronic supplementary material

Below is the link to the electronic supplementary material.

Supplementary material 1 (DOCX 20 kb)
