# Supplementary material for: Pasireotide LAR maintains inhibition of GH and IGF-1 in patients with acromegaly for up to 25 months: results from the blinded extension phase of a randomized, double-blind, multicenter, Phase III study
Source: Pituitary. 2014 Aug 8;18(3):385–94. doi: 10.1007/s11102-014-0585-6 (PMC4424273; doi:10.1007/s11102-014-0585-6)
Supplement: Supplementary file 1 — Supplementary material 1 (DOCX 20 kb) [file 11102_2014_585_MOESM1_ESM.docx]

**Supplementary Appendix**

**Pasireotide LAR maintains inhibition of GH and IGF-1 in patients with acromegaly for up to 25 months: results from the blinded extension phase of a randomized, double-blind, multicenter, Phase III study**

**Table of contents**

Tumor volume assessment 2

**Tumor volume assessment**

The intra-observer variability of the blinded central reader was assessed independently by a third-party organization with experience in radiological measurement, repeatability and reproducibility. The central reader measured pituitary tumor volumes in patients undergoing treatment, then re-read the images of 10 randomly selected patients from a total of 29 time points, in a blinded manner and not earlier than 3 weeks after the original reading. Variability analysis was performed on a time-point-by-time-point and case basis. For the time-point-by-time-point analysis, the absolute and percentage differences between the volumes rendered at the first and secondary reviews were determined at each time point. For the case analysis, the percentage volume change at each post-baseline time point in comparison with baseline was calculated for the first and secondary reviews and compared against each other. The results of the analysis were consistent, reproducible and, in most cases, unaffected by complex tumor anatomy, small absolute tumor size and/or poor image quality. The results of the case analysis showed percentage change differences in excess of 10% in 26% of the cases, reflecting a reasonable level of reading consistency. The variability in central reader measurements falls well within the 20% volume change threshold specified in the study as indicating a significant change in tumor volume.
